# The prolonged effect of film mulch and P application on lucerne forage yield in a semiarid environment

**DOI:** 10.3389/fpls.2023.1331704

**Published:** 2023-12-11

**Authors:** Meng Kong, Yan-Jie Gu, Cheng-Long Han, Xiao-Peng Shi, Jing Kang, Kadambot H. M. Siddique, Feng-Min Li, Zi-Qiang Yuan

**Affiliations:** ^1^ Shanxi Province Key Laboratory of Sustainable Dryland Agriculture, Shanxi Institute of Sustainable Dryland Agriculture, Shanxi Agricultural University, Taiyuan, Shanxi, China; ^2^ Key Laboratory of Sustainable Dryland Agriculture (Co-construction by Ministry of Agriculture and Rural Affairs and Shanxi Province), Shanxi Agricultural University, Taiyuan, Shanxi, China; ^3^ State Key Laboratory of Herbage Improvement and Grassland Agroecosystems, College of Ecology, Lanzhou University, Lanzhou, Gansu, China; ^4^ State Key Laboratory of Plateau Ecology and Agriculture, Department of Grassland Science, College of Agricultural and Husbandry, Qinghai University, Xining, Qinghai, China; ^5^ Institute of Agriculture, The University of Western Australia, Perth, WA, Australia; ^6^ Jiangsu Collaborative Innovation Center for Modern Crop Production co-sponsored by Province and Ministry, College of Agriculture, Nanjing Agricultural University, Nanjing, China

**Keywords:** film mulch, phosphorus application, legume forage, soil water deficit, soil fertility, loess plateau

## Abstract

**Introduction:**

Limited water and soil phosphorus (P) availability often hampers lucerne productivity in semiarid regions. Plastic film mulch and P application typically enhance young lucerne (2–3 years) productivity by increasing soil water use and P availability. However, the prolonged impact of film mulch and P application on lucerne productivity as the stand ages remains unclear.

**Methods:**

This study conducted a 9-year field experiment on the semiarid Loess Plateau to investigate how film mulch and P application affect lucerne forage yield, soil water content, and soil fertility. The field experiment used a split-plot design with randomized blocks, in which the whole plots were with (M1) and without plastic film mulch (M0), and the split plots were four P rates (0 (P0), 9.7 (P1), 19.2 (P2), and 28.8 (P3) kg P ha^−1^).

**Results and discussion:**

The M1 treatment produced significantly higher lucerne forage yields than the M0 treatment during the first five years, but the yield-increasing effect of film mulch gradually diminished over time, with no effect in Years 6–8, and lower yields than the M0 treatment in Year 9. Phosphorus fertilization significantly increased forage yield after Year 3 in the M0 treatment, but only in Years 3–5 in the M1 treatment. In Years 2–5, film mulch significantly increased soil organic carbon, total nitrogen (N), inorganic N, and microbial biomass carbon in P0, P1, and P2 but not in P3. However, in Years 7–9, film mulch significantly decreased soil available potassium (K), organic carbon mineralization, lucerne density, and shoot K concentration, but did not reduce soil N and P availability at any level P of application. Moreover, plastic film mulch significantly increased the soil water content at 0–300 cm deep from Year 7 onwards. In conclusion, film mulch ceased to enhance lucerne production beyond year 6, which could not be attributed to soil water content, N or P availability but was partially associated with reduced soil K availability. Consequently, future research should focus on soil K availability, and K addition should be considered after five years in lucerne pastures mulched with plastic film in semiarid areas.

## 1 Introduction

Lucerne (*Medicago sativa* L.) is a valuable forage crop known for its excellent forage yield, high protein content, environmental adaptability, and ecological benefits (Testa et al., 2011; Wang et al., 2021; Feng et al., 2022). It is widely cultivated in semiarid regions, where it helps reduce soil erosion, enhance soil fertility, and address forage shortages in animal husbandry (Ali et al., 2021; Fang et al., 2021). However, the growth of lucerne in semiarid areas is often limited by low soil water and phosphorus (P) availability, resulting in low forage yields (Jiang et al., 2006; Fan et al., 2016; Wang et al., 2021). Plastic film mulch is commonly used to increase crop productivity in semiarid regions, promoting precipitation infiltration into soils, reducing soil water evaporation, and improving soil water content and nutrient availability (Liu et al., 2009; Zhu et al., 2018). In addition, P fertilizer application is a powerful means of improving soil P availability and promoting plant growth in dryland environments (Berg et al., 2007; Fan et al., 2011). Thus, combining plastic film mulch and P application provides a means to improve lucerne productivity in semiarid areas (Gu et al., 2018).

While research has shown that plastic film mulch and P application can increase lucerne forage yield (Berg et al., 2005; Jia et al., 2006a; Wang et al., 2015), these studies mainly focused on lucerne at the young stage (2–3 years). Lucerne is a perennial plant that can grow for many years, with its productivity changing with stand age (Wang et al., 2008; Fan et al., 2011; Wang et al., 2021). Therefore, understanding the effects of prolonged plastic film mulch and P application on forage production requires studies of lucerne at different stages of maturity to comprehensively evaluate the effects of plastic film mulch and P fertilization on its forage production and to offer insights into optimizing lucerne pasture management for improved forage yields. However, little information is available on the prolonged impact of plastic film mulch and P application on lucerne production and persistence in semiarid areas. Furthermore, the key factors affecting its yield-increasing effects with increasing stand age remain inadequately explored.

Adequate use of the available water is crucial for sustained lucerne yield (Huang et al., 2018; Wang et al., 2021). Although plastic film mulch can increase soil water content in the upper soil layer by increasing rainwater infiltration into soils and decreasing soil water evaporation in semiarid regions, plastic film mulch and P application could intensify deep soil water use through enhanced lucerne growth at the young stage (Jia et al., 2006b; Fan et al., 2011; Gu et al., 2018), which may have negative effects on the soil water balance and lucerne productivity with increasing stand age. Furthermore, due to its deep-rooted nature and high water consumption (Ali et al., 2021), lucerne can cause deep soil drying and increase soil water storage deficits as its root system extends with stand age (Wang et al., 2008; Ali et al., 2021). The yield decline of aged lucerne fields may be associated with reduced soil water storage (Wang et al., 2021). However, the temporal dynamics of soil water storage in lucerne fields following film mulch and P application and the effect on the forage yield remain poorly understood.

Soil fertility is a major factor influencing lucerne productivity (Feng et al., 2022). Previous studies have shown that lucerne forage yield positively correlates with soil inorganic nitrogen (IN), available P (AP), and available potassium (AK) contents over a large scale (Xie et al., 2016; Feng et al., 2022). Fertilization with P and K enhance lucerne nitrogen (N) fixation, carbohydrate synthesis, and yield (Berg et al., 2007; Jungers et al., 2019), while appropriate application of N fertilizer significantly improves lucerne productivity in soils with low available N (Fan et al., 2016). While lucerne can fix free atmospheric N and enhance soil organic carbon (SOC) and total nitrogen (STN) concentrations through its deep root system and N-fixing capacity (Fang et al., 2021; Wang et al., 2021), continuous harvests eventually deplete soil IN, AP, and AK (Jia et al., 2006b; Xie et al., 2016; Yuan et al., 2016). Therefore, despite prior reports indicating film mulch and P application can improve soil fertility in young lucerne stands (Kou et al., 2011; Fan et al., 2016), large amounts of nutrients are removed from the soil due to increased forage yield, and this phenomenon will gradually increase as the stand ages, which may eventually lead to soil nutrient imbalances. However, the prolonged impact of film mulch and P application on soil nutrient content remains largely explored. Furthermore, changes in soil nutrient availability might yield insights into the sustained yield-increasing effects of film mulch and P application. However, whether the changes in soil nutrients under film mulch and P application can consistently sustain forage yields as lucerne ages remains unknown.

In summary, the substantial removal of soil nutrients and potential exacerbation of soil water deficits in young lucerne stands could hinder future lucerne growth demands, consequently impeding productivity. This scenario could be the crucial limiting factor governing the yield-increasing effects of film mulch and P application. Therefore, we hypothesized that the yield-increasing effects of film mulch and P application gradually decline with increasing stand age. In this study, we present the results of a 9-year lucerne field experiment conducted in a semiarid region to investigate the effects of prolonged film mulch and P application on lucerne yield, soil water content, and soil fertility dynamics. Our objectives were to: (1) determine the yield effects of film mulch and P application on lucerne in stands of different ages; (2) examine the effects of film mulch and P application on soil water storage and nutrient content as lucerne stands age; and (3) identify the key factors associated with changes in the yield-increasing effects of film mulch and P application on lucerne with increasing stand age. The findings of this study should contribute to the development of sustainable, high-yielding lucerne pastures while advancing animal husbandry in semiarid regions.

## 2 Materials and methods

### 2.1 Study site

The field study was conducted from 2011 to 2019 at the Dryland Agro-Ecosystem Research Station of Lanzhou University, Zhonglianchuan village, Yuzhong county, Gansu province, China (36°02′ N, 104°25′ E; 2,400 m above sea level). The climate is medium temperate and semiarid, with a mean annual air temperature of 6.5°C. The mean annual precipitation from 2002 to 2019 was 333 mm, with about 60% occurring between June and September (**Figure S1**). Precipitation in each year from 2011 to 2019 was 281, 415, 367, 421, 297, 285, 350, 462, and 416 mm, respectively (**Figure S1**). The soil type is Calcic Kastanozem, with 12.3% sand, 66.9% silt, and 20.8% clay. The initial (2011) soil properties (0–20 cm deep) were: 0.196 g g^−1^ maximum field water-holding capacity, 0.047 g g^−1^ permanent wilting coefficient (Shi et al., 2003), pH 8.8, 5.28 g kg^−1^ organic carbon; 0.34 g kg^−1^ total N, 0.65 g kg^−1^ total P, and 4.60 mg kg^−1^ available P.

### 2.2 Experimental design

The experiment had a split-plot design with three randomized blocks. The main plots were plastic film mulching with ridge–furrow planting (M1) and no mulching with flat planting (M0). The subplots included four P rates: 0 (P0), 9.7 (P1), 19.2 (P2), and 28.8 (P3) kg P ha^−1^. The experiment started in June 2011 and ended in October 2019. Before sowing, urea (34.5 kg N ha^–1^) was spread evenly over each plot and plowed into the soil as a base fertilizer. The applied P fertilizer was calcium superphosphate (CaP_2_H_4_O_8_), with the pure P content determined each year (**Table S1**). From 2012 to 2019, only P fertilizer was applied. In 2012 and 2013, P fertilizer was applied between rows (M0) or on ridges (M1) to protect the young lucerne roots. From 2014 to 2019, P fertilizer was applied alternately to both sides of each row to about 10 cm depth using a triangular hoe to ensure its proximity to the adult lucerne root system.

The ridge width and height in the M1 treatment were 30 cm and 15 cm, respectively. The furrows were V-shaped to promote rainwater infiltration into the soil, with the lucerne grown within the furrows. The ridge and furrow structure was formed before sowing using a tractor, and the ridge was covered with transparent polyethylene film (0.008 mm thick, 40 cm wide, Lanzhou Jintudi Plastic Products Co., Ltd., Lanzhou, China). On 29 June, 2011, ‘Longzhong’ lucerne seeds were sown at 15 kg ha^–1^ in strips at a depth of about 4 cm with a row spacing of 30 cm in all treatments, as per the typical lucerne planting practice in the region. After lucerne regreening in April each year, the previous year’s film was removed with a rake, followed by P fertilizer application, and new plastic film was laid on the original ridge by hand, burying the edges under the soil. Each plot was 30 m^2^ (10 × 3 m) from 2011 to 2016. From 2017 to 2019, without altering plot location, each plot was subdivided into two 15 m^2^ (5 × 3 m) plots, with one of these randomly selected to continue the experiment. After excluding the edge area, the plot’s sampling area was 8.4 m^2^ from 2017 to 2019, which did not affect the sampling process. Thus, reducing the plot size did not affect the experiment.

### 2.3 Sampling and measurements

#### 2.3.1 Lucerne forage yield

The lucerne was harvested twice each year except in 2011 when it was harvested only once in mid-October. Harvest occurred at the whole flowering stages (mid-July and mid-October) from 2012 to 2016 and at the early flowering stages (late June and late August) from 2017 to 2019. Yang (2013) reported no difference in lucerne forage yield between the early flowering stage and the end of the flowering period in the rainfed agricultural areas of the Loess Plateau, but higher nutrient contents during early flowering. From 2011 to 2016, after excluding the edge areas of each plot, three 2 m long lucerne rows were sampled at random (total 1.8 m^2^). Due to the decline in lucerne density over time, after excluding the edge areas in 2017–2019, the remaining part of the plot was used as the yield measurement area (4 × 2.1 m, 8.4 m^2^). At harvest, the fresh weight of lucerne in the yield measurement area was recorded, and 200–500 g of fresh lucerne was oven-dried to constant weight at 75°C to calculate the biomass moisture content. Lucerne forage yield per hectare with dry weight (DY) was calculated as:


(1) $$\text{DY}=\frac{FY}{A}\times 10000\times \left(1-MC\right)$$

where FY is the fresh weight per yield measurement area (kg), A is the yield measurement area (m^2^, 1.8 m^2^ in 2011–2016 and 8.4 m^2^ in 2017–2019), and MC is the biomass moisture content (%).

The annual forage yield was the sum of the two harvests. Three 4 m long lucerne rows were randomly selected within each plot before the first harvest to determine plant height in Years 2–9 and stand density (the number of shoots per unit area) in Years 4–9. After each harvest, the remaining lucerne was mown and removed from the plots by local farmers.

The annual average lucerne yield was calculated to represent the continuous high yield level:


(2) $$\text{AAYn }\left(\text{n}=1,2,3,\ldots ,9\right)=\frac{\sum_{i=1}^{n}Yi}{n}$$

where AAYn is the average lucerne yield of n years (t ha^–1^), Yi is the yield for a given year, and n is the year number.

The yield-increasing effect of film mulch or P application (E_treatment_, %) was calculated as:


(3) $$E_{treatment}=\frac{Y_{treatment}-Y_{ck}}{Y_{ck}}\times 100\%$$

where Y_treatmen_t is the forage yield under film mulch or P application treatment (t ha^–1^), and Y_ck_ is the forage yield in the M0 or P0 treatment (t ha^–1^).

The net income was calculated as income minus input costs. Farmers in this area do not consider labor costs in traditional lucerne production, so the annual inputs included the purchase costs of the phosphate fertilizer and plastic film and the labor costs of forming ridges and covering the ridges with film. The annual cost of the M1 treatment was 1,389 RMB Yuan ha^–1^ higher than that of the M0 treatment due to the cost of plastic film, forming the ridges, and covering the ridges with film. In the first year, the purchase costs of lucerne seeds (35 RMB Yuan kg^−1^) and urea (2.0 RMB Yuan kg^−1^) were added to the input costs. The average price of phosphate fertilizer was 1.125 Yuan kg^–1^. Income was calculated as the forage yield multiplied by the average local lucerne price (0.5 RMB Yuan kg^−1^).

#### 2.3.2 Shoot N, P, and K concentrations

For each plot, approximately 15–20 representative lucerne plants were oven-dried at 75°C for elemental analysis. The oven-dried forage samples from each harvest in each year were ground, and subsamples of approximately 200 mg were digested with H_2_O_2_-H_2_SO_4_ to determine their N concentration using the Kjeldahl method, P concentration using molybdenum-antimony anti-spectrophotometry, and K concentration using digital flame photometry (Thomas et al., 1967).

#### 2.3.3 Soil water content

Soil water content of each plot was measured at 20 cm intervals to 500 cm depth at the beginning of the growing season (April) from 2012 to 2019 using a hand-held soil auger (5 cm diameter). Soil samples were oven-dried at 105°C to determine the soil water content. Soil water storage (SWS, mm) was calculated as:


(4) SWS=WC×BD×H×0.1

where WC, BD, and H are soil water content (%), bulk density (g cm^–3^), and layer thickness (cm), respectively. The SWS change was calculated as the annual SWS minus the SWS at pre-sowing (June 2011).

#### 2.3.4 Soil fertility

Because the soil nutrients are concentrated in the 0–20 cm soil layer in the semiarid Loess Plateau of China (Fan et al., 2016; Wang et al., 2021), we collected soil from the upper 20 cm to determine soil fertility levels. From 2012 to 2019, three subsoil samples from the ridge and three from the furrow were randomly taken in each plot at the final harvest using a soil auger (20 cm depth, 5 cm diameter), and then bulked to form a composite soil sample for analysis. Soil samples were collected from the soil profiles at 0–20, 20–60, and 60–100 cm depth in the same manner in each plot in April 2019 to examine soil profile fertility responses to film mulch and P application. Soil fertility, including light fraction organic carbon (LFOC), total P (STP), SOC, STN, AP, IN, AK, and microbial biomass carbon (MBC), were determined using various methods: density fractionation method (Jiang et al., 2006), molybdenum-antimony colorimetric method (Lu, 2000), Walkley–Black method with a correction coefficient of 1.33 (Lu, 2000), Kjeldahl analysis (Lu, 2000), Olsen-P method (Olsen et al., 1954), flow injection analyzer (Skalar Analytical B.V., Breda, Netherlands) after extraction with 0.5 mol L^–1^ K_2_SO_4_, digital flame photometer (M410, Sherwood Scientific Ltd., Cambridge, UK) after extraction with 1.0 mol L^–1^ CH_3_COONH_4_ (Lu, 2000), and the chloroform fumigation‒extraction method with an efficiency constant of 0.45 (Jenkinson, 2004), respectively.

#### 2.3.5 SOC mineralization

Soils were sampled from the top 20 cm in each plot in July 2018 to measure SOC mineralization. Two treatments were established to test the effect of soil K availability on SOC mineralization: control soil without K amendment (K0) and soil amended with KCl at 30 mg K kg^–1^ soil^–1^ (K1). All soil samples were adjusted to 60% water-holding capacity and then pre-incubated for a week in the dark (25°C). SOC mineralization was determined by measuring the CO_2_ released during a 91-day incubation experiment at 25°C. For each soil sample, 100 g was placed in a 500 mL jar (8 cm diameter) with a beaker containing 10 mL of 1 mol L^–1^ NaOH to absorb CO_2_ and another beaker containing 10 mL distilled water to maintain soil water content. Soil respiration was measured on days 2, 6, 13, 27, 41, 55, 69, and 91. After incubation, the NaOH in the vials was titrated with 0.05 M hydrochloric acid to quantify the amount of evolved CO_2_. The cumulative SOC mineralized was the total amount of evolved CO_2_ over the 91 days.

### 2.4 Statistical analysis

The statistical analyses were performed using GenStat v. 18.1 (VSN International, Hemel Hempstead, UK). The effects of film mulch and P application and their interaction were evaluated using a randomized complete block of the split-plot design for variables including forage yield, plant height, the number of shoots per unit area, shoot nutrient concentrations, soil water storage, fertility, SOC mineralization, and economic benefit. Independent sample t-tests were used to examine the effects of K addition on SOC mineralization. Significance differences were determined at *p* ≤ 0.001, *p* ≤ 0.01, *p* ≤ 0.05, and *p* ≤ 0.1. Polynomial regression analyses were used to assess the relationships between the yield-increasing effects of film mulch and stand age. There was no significant relationship between the yield-increasing effect of P application and stand age in either treatment, so the relationship between the yield-increasing effect of P application and stand age is presented using spline curves based on the mean. The correlation between lucerne forage yield and soil properties was performed using Pearson’s correlation coefficients. The figures were drawn using Origin Pro 2015 (Origin Lab, Massachusetts, USA).

## 3 Results

### 3.1 Lucerne forage yield and its components

**Figure 1:**
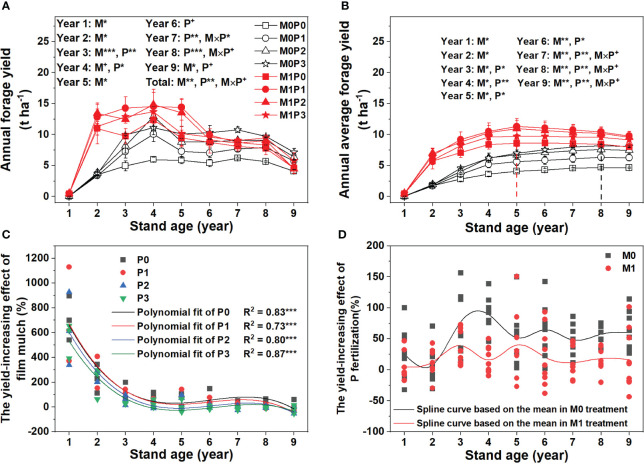
Dynamic changes in **(A)** annual lucerne forage yield and **(B)** annual average forage yield, and the relationships between lucerne stand age and the yield-increasing effect of **(C)** film mulch and **(D)** P fertilization for no film mulch (M0) and film mulch (M1) at four P levels [0 (P0), 9.7 (P1), 19.2 (P2), and 28.8 (P3) kg P ha^−1^ year^–1^] in Gansu Province, China. ***p ≤ 0.001, **p ≤ 0.01, * *p* ≤ 0.05, and + *p* ≤ 0.1. Data are mean ± standard error (n = 3). No significant relationship occurred between the yield-increasing effect of P application and stand age in either treatment, so a spline curve based on the mean was used to illustrate this relationship.

The M1 treatment significantly increased lucerne forage yield in Years 1–5, had no effect in Years 6–8, and decreased lucerne forage yield in Year 9, relative to the M0 treatment (**Figure 1A**). In Years 1 and 2, P application did not significantly affect lucerne forage yield. In the M0 treatment, P application significantly increased lucerne forage yield in Years 3–9, with the P1, P2, and P3 treatments averaging 39.0%, 66.0%, and 81.4% higher forage yields than the P0 treatment during this time, respectively (**Figure 1A**). In the M1 treatment, P application significantly increased lucerne forage yield in Years 3–5 only, with the P1, P2, and P3 treatments averaging 36.8%, 29.2%, and 16.4% higher forage yields, respectively, than the P0 treatment during this time (**Figure 1A**). The annual average forage yield in the M1 treatment peaked in Year 5, three years earlier than the M0 treatment (**Figure 1B**). The forage yield-increasing effect of film mulch gradually decreased with stand age, becoming negative in Year 9 (–12.9%) (**Figure 1C**). No significant relationship occurred between the yield-increasing effect of P application and stand age in either treatment (**Figure 1D**).

**Figure 2:**
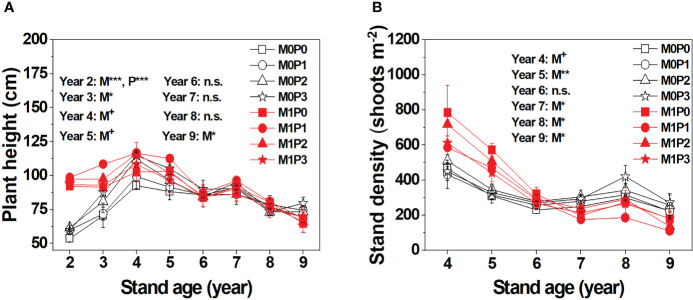
Dynamic changes in lucerne **(A)** plant height and **(B)** stand density (shoots m^–2^) of the first cut lucerne for no film mulch (M0) and film mulch (M1) at four P levels [0 (P0), 9.7 (P1), 19.2 (P2), and 28.8 (P3) kg P ha^−1^ year^–1^] in Gansu Province, China. ***p ≤ 0.001, **p ≤ 0.01, *p ≤ 0.05, and + *p* ≤ 0.1. Omitted contrasts and those shown with an abbreviation (n.s.) were not significant (*p* > 0.1). Data are mean ± standard error (n = 3).

The M1 treatment had significantly taller lucerne plants than M0 in Years 2–5, no significant differences in Years 6–8, and significantly shorter plants than M0 in Year 9 (**Figure 2A**). Fertilization with P significantly increased lucerne plant height only in Year 2. Film mulch significantly increased the number of shoots per unit area in Years 4–5, had no effect in Year 6, and decreased the number of shoots per unit area in Years 7–9 (**Figure 2B**). Fertilization with P did not affect the number of shoots per unit area (**Figure 2B**).

### 3.2 Shoot N, P, and K concentrations

**Figure 3:**
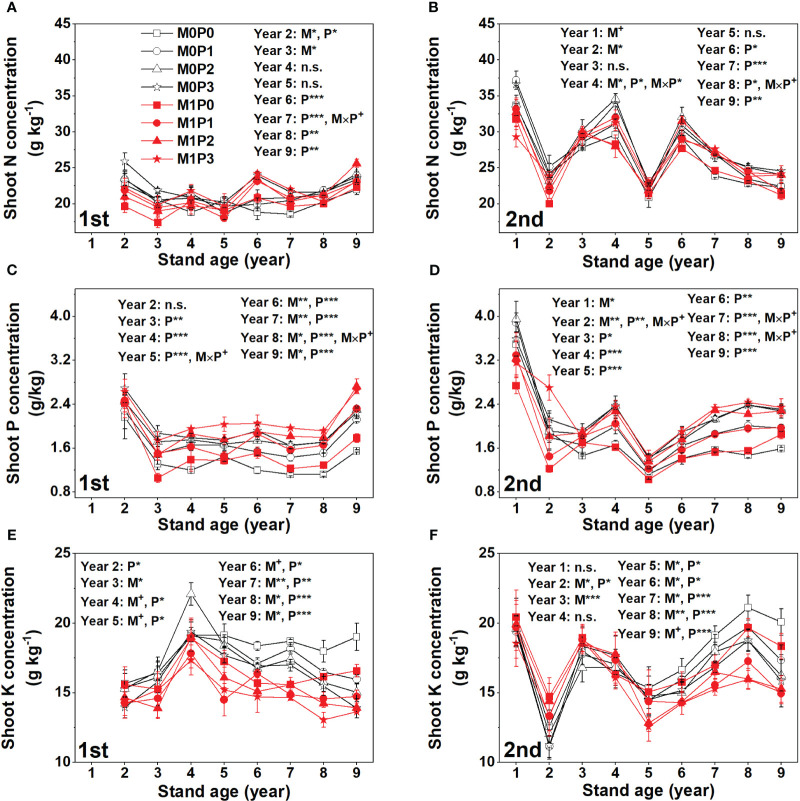
Dynamic changes in shoot **(A, B)** N, **(C, D)** P, and **(E, F)** K concentrations of the **(A, C, E)** first and **(B, D, F)** second cut lucerne for no film mulch (M0) and film mulch (M1) at four P levels [0 (P0), 9.7 (P1), 19.2 (P2), and 28.8 (P3) kg P ha^−1^ year^–1^] in Gansu Province, China. ***p ≤ 0.001, **p ≤ 0.01, *p ≤ 0.05, and + *p* ≤ 0.1. Omitted contrasts and those shown with an abbreviation (n.s.) were not significant (*p* > 0.1). Data are mean ± standard error (n = 3).

The M1 treatment had significantly higher lucerne shoot N concentrations than M0 in Years 1–3 only (**Figures 3A, B**). Fertilization with P significantly increased shoot N concentration in Years 6–9 (**Figures 3A, B**). Film mulch significantly decreased shoot P concentration in the second cut lucerne in Years 1–2, increased the shoot P concentration in the first cut lucerne in Years 6–9, and had no effect in other years (**Figures 3C, D**). Fertilization with P significantly increased shoot P concentration during the experimental period (except for Year 1 and the first cut lucerne in Year 2) (**Figures 3C, D**). Film mulch significantly increased shoot K concentration in the second cut lucerne in Years 2–3 and decreased shoot K concentration in the first cut lucerne in Years 3–9 and the second cut lucerne in Years 5–9 (**Figures 3E, F**). Overall, P application significantly decreased shoot K concentrations (**Figures 3E, F**).

### 3.3 Soil water

**Figure 4:**
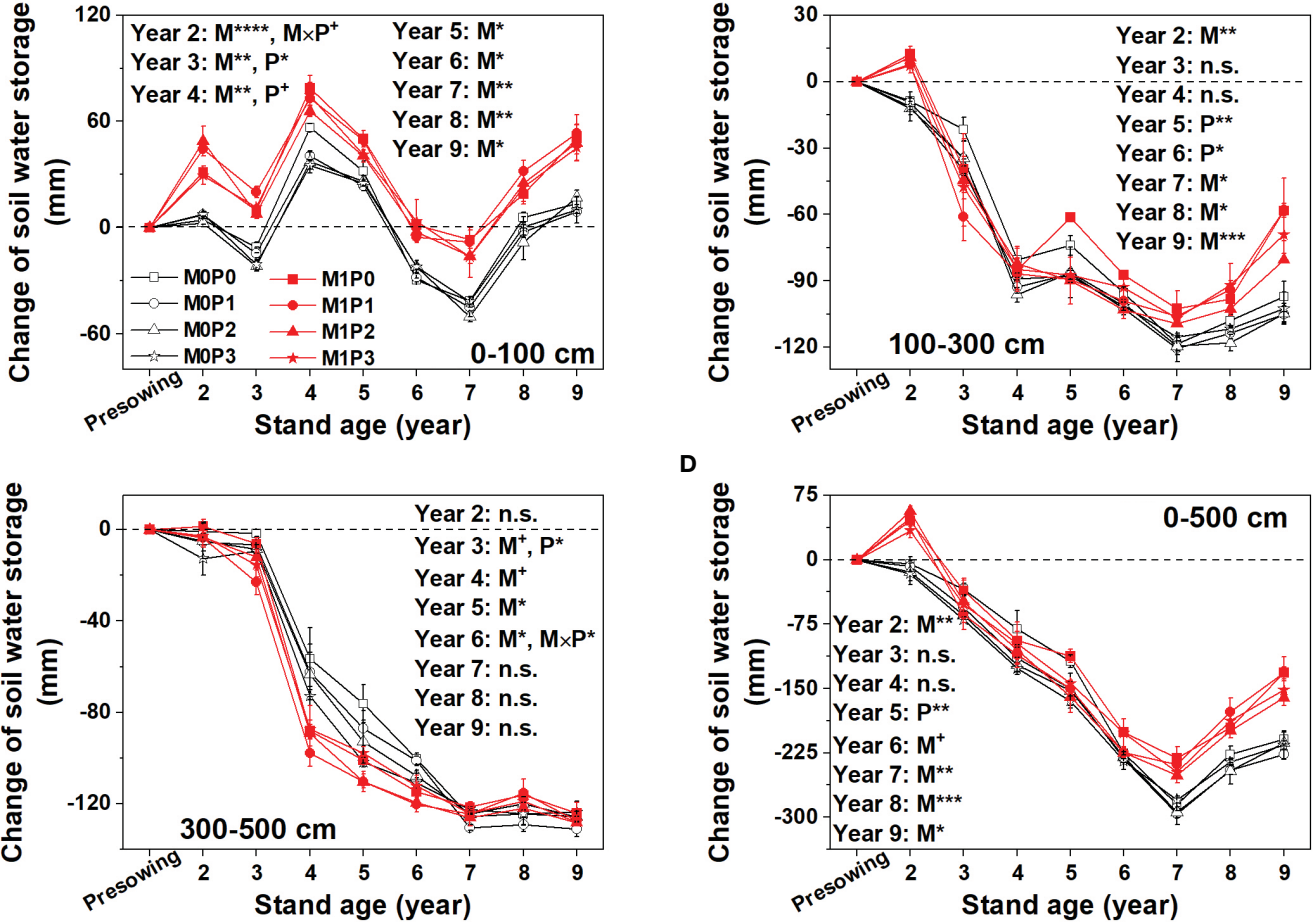
Changes in soil water storage (annual soil water storage minus soil water storage before lucerne sowing) with time in the **(A)** upper 100 cm, **(B)** 100–300 cm, **(C)** 300–500 cm, and **(D)** 0–500 soil profile for no film mulch (M0) and film mulch (M1) at four P levels [0 (P0), 9.7 (P1), 19.2 (P2), and 28.8 (P3) kg P ha^−1^ year^–1^] at the beginning of the growing season in Gansu Province, China. ***p ≤ 0.001, **p ≤ 0.01, *p ≤ 0.05, and + *p* ≤ 0.1. Omitted contrasts and those shown with an abbreviation (n.s.) were not significant (*p* > 0.1). Data are mean ± standard error (n = 3).

The M1 treatment had significantly higher 0–100 cm SWS than the M0 treatment during the experimental period (**Figures 4A**, **S3**). Film mulch significantly increased the 100–300 cm SWS in Years 2 and 7–9 (**Figure 4B**). Application with P significantly decreased the 100–300 cm SWS in Years 5 and 6 (**Figure 4B**). In Year 9, the M1 treatment had 25.5% higher average 100–300 cm SWS than M0. The 300–500 cm SWS decreased sharply in Year 4, then decreased slowly, reaching its lowest value in Year 7 before stabilizing (**Figure 4C**). Film mulch significantly decreased the 300–500 cm SWS in Years 3–6. The change in SWS in the 0–500 cm soil profile decreased with planting Years 2–7 and then increased. Film mulch significantly increased 0–500 cm SWS in Years 2 and 6–9, and P application significantly decreased 0–500 cm SWS in Year 5 (**Figure 4D**).

### 3.4 Soil fertility

**Figure 5:**
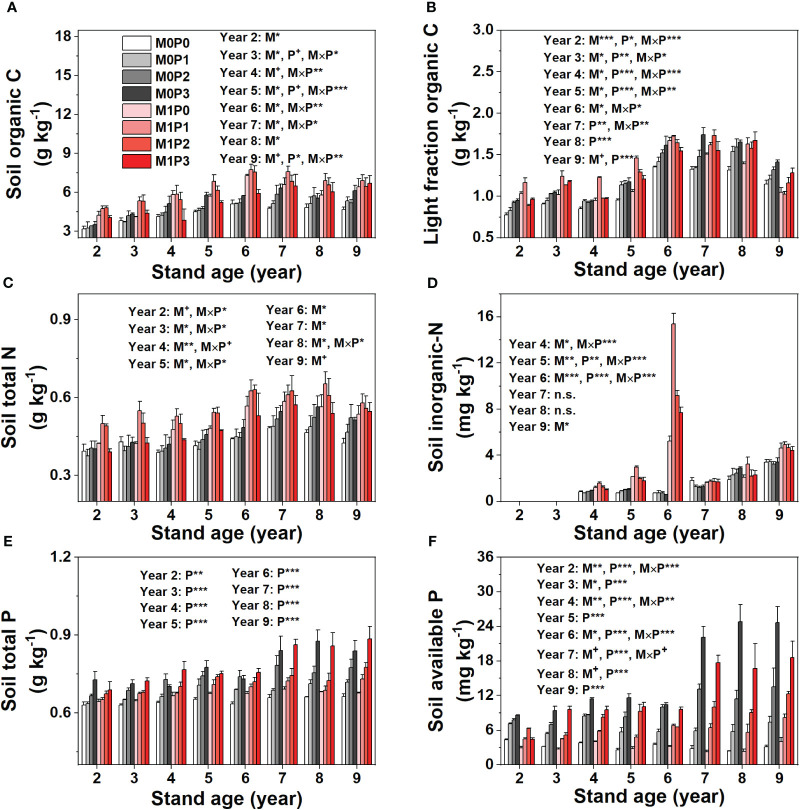
Dynamic changes in **(A)** soil organic C, **(B)** light fraction organic C, **(C)** total N, **(D)** inorganic N, **(E)** total P, and **(F)** available P for no film mulch (M0) and film mulch (M1) at four P levels [0 (P0), 9.7 (P1), 19.2 (P2), and 28.8 (P3) kg P ha^−1^ year^–1^] at the end of the growing season in Gansu Province, China. ***p ≤ 0.001, **p ≤ 0.01, *p ≤ 0.05, and + *p* ≤ 0.1. Omitted contrasts and those shown with an abbreviation (n.s.) were not significant (*p* > 0.1). Data are mean ± standard error (n = 3).

The M1 treatment had significantly more SOC and STN than the M0 treatment in the P0, P1, and P2 treatments but not in P3 in Years 2–9 (**Figures 5A, C**). The SOC and STN increased significantly with increasing P rate in the M0 treatment but increased and then decreased in the M1 treatment, reaching the highest level under P1 (**Figures 5A, C**). Film mulch significantly increased soil LFOC in Years 2–6 except in P3 treatment, had no effect in Years 7–8, and decreased soil LFOC in Year 9 (**Figure 5B**). In Years 2–7, with increasing P rate, LFOC increased in the M0 treatment but increased and then decreased in the M1 treatment. In Years 8–9, LFOC increased with increasing P level in both treatments (**Figure 5B**). Film mulch significantly increased soil IN in Years 4–6 and Year 9 but had no effect in Years 7–8 (**Figure 5D**).

**Figure 6:**
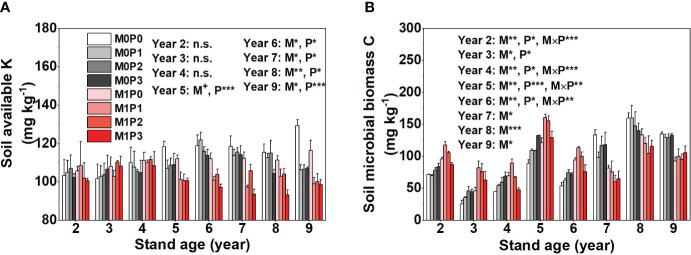
Dynamics in soil **(A)** available K and **(B)** microbial biomass C for no film mulch (M0) and film mulch (M1) at four P levels [0 (P0), 9.7 (P1), 19.2 (P2), and 28.8 (P3) kg P ha^−1^ year^–1^] at the end of the growing season in Gansu Province, China. ***p ≤ 0.001, **p ≤ 0.01, *p ≤ 0.05, and + *p* ≤ 0.1. Omitted contrasts and those shown with an abbreviation (n.s.) were not significant (*p* > 0.1). Data are mean ± standard error (n = 3).

Fertilization with P significantly increased STP and AP with increasing lucerne stand age and increasing P application rate (**Figures 5E, F**), while film mulch did not affect STP. The M1 treatment had lower soil AP than M0, except in Year 5 and 9 (**Figures 5E, F**). Film mulch and P application significantly decreased soil AK content in Years 5–9 but had no effect in Years 2–4 (**Figure 6A**). In April 2019, all treatments showed low soil IN (<2.8 mg kg^−1^) and AP (<1.8 mg kg^−1^) concentrations in the 20–100 m soil layers (**Figure S4**).

### 3.5 Soil MBC and SOC mineralization

**Figure 7:**
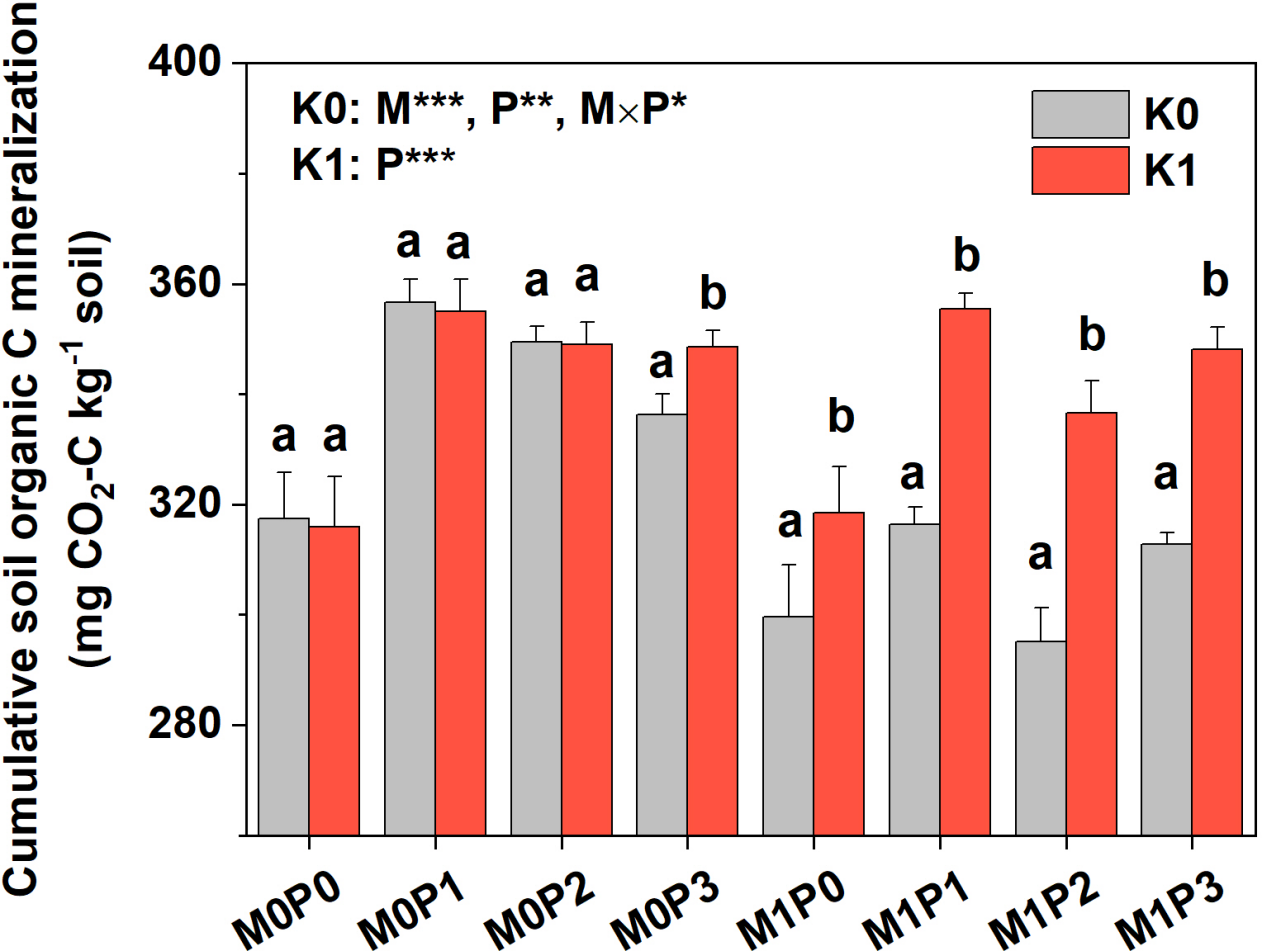
Cumulative soil organic C mineralization for no film mulch (M0) and film mulch (M1) at four P levels [0 (P0), 9.7 (P1), 19.2 (P2), and 28.8 (P3) kg P ha^−1^ year^–1^] during a 91-day incubation experiment without (K0) or with K addition (K1) in July 2018. ***p ≤ 0.001, **p ≤ 0.01, and * *p* ≤ 0.05. Different lowercase letters between K0 and K1 indicate a significant difference at *p* < 0.05. Data are mean ± standard error (n = 3).

Film mulch significantly increased soil MBC in Years 2–6 before it then decreased (**Figure 6B**). Fertilization with P had a significant effect on soil MBC in Years 2–6, after which no significant effect was detected (**Figure 6B**). Soil MBC increased significantly with increasing P rate in the M0 treatment but increased and then decreased in the M1 treatment in Years 2–6 (**Figure 6B**). The M1 treatment had significantly lower cumulative SOC mineralization than the M0 treatment (**Figure 7**). In the M0 treatment, P application significantly increased cumulative SOC mineralization, with the P1, P2, and P3 treatments being 15.8%, 11.7%, and 7.5% higher than the P0 treatment, respectively. In the M1 treatment, P application did not affect cumulative SOC mineralization (**Figure 7**). Soil amended with K showed significantly increased cumulative SOC mineralization in the M1 treatment. In contrast, soil amended with K in the M0 treatment showed no effect on cumulative SOC mineralization in P0, P1, or P2, but significantly increased it in P3 (**Figure 7**).

### 3.6 Correlations between forage yield and soil properties

**Figure 8:**
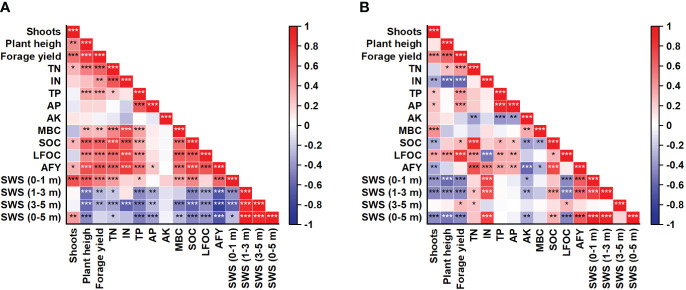
Correlations between lucerne forage yield and soil properties from Years **(A)** 2–5 and **(B)** 7–9. TN, total nitrogen; IN, inorganic N; TP, total P; AP, available P; AK, available K; MBC, microbial biomass carbon; SOC, soil organic C; LFOC, light fraction organic C; CFY, cumulative forage yield; SWS, soil water storage. ***p ≤ 0.001, **p ≤ 0.01, *p ≤ 0.05.

Significant positive correlations were observed between forage yield and plant height, shoot number, SOC, LFOC, TN, and TP during Years 2–9 (**Figure 8A, B**). Forage yield was positively correlated with the 0–100 cm SWS during Years 2–5 but was negatively correlated with SWS in the 100–300 cm and 300–500 cm layers during Years 2–5 and SWS in the 0–100, 100–300, and 0–500 cm layers during Years 7–9 (**Figures 8A, B**). Soil AK was positively correlated with cumulative forage yield and MBC during Years 7–9 (**Figures 8A, Bb**).

### 3.7 Economic benefit

**Figure 9:**
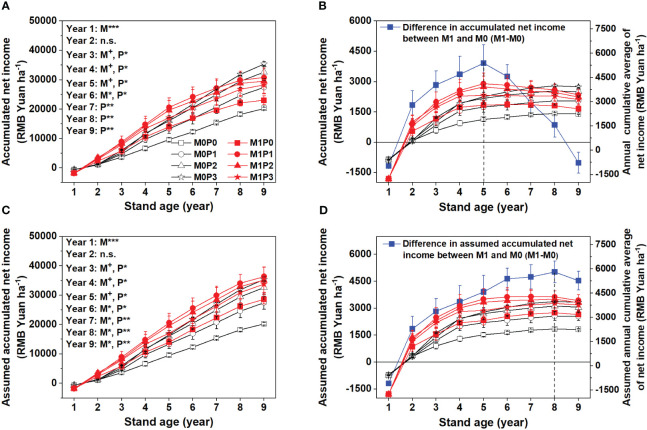
Accumulated net income **(A)**, difference in accumulated net income between M1 and M0 and annual cumulative average of net income **(B)**, assumed accumulated net income (film replacement stops in Year 6 and yields remained unchanged **(C)**, and difference in assumed accumulated net income between M1 and M0 and assumed annual cumulative average of net income **(D)** for no film mulch (M0) and film mulch (M1) at four P levels [0 (P0), 9.7 (P1), 19.2 (P2), and 28.8 (P3) kg P ha^−1^ year^–1^] in Gansu Province, China. ***p ≤ 0.001, **p ≤ 0.01, *p ≤ 0.05, and + *p* ≤ 0.1. Omitted contrasts and those shown with an abbreviation (n.s.) were not significant (p > 0.1). Data are mean ± standard error (n = 3).

Film mulch significantly increased the cumulative net income in years 3–6 (**Figure 9A**). The cumulative net income difference between the M1 and M0 treatments (M1–M0) peaked peaking at 3,911 RMB Yuan ha^–1^ in Year 5. However, in Years 7–9, the beneficial effect declined and became statistically insignificant (**Figures 9A, B**). From Year 3 onwards, P application significantly increased cumulative net income. The highest cumulative net income occurred in the M1P1 treatment in Years 1–7 and the M0P3 treatment in Years 8 and 9 (**Figure 9A**).

To explore the various strategies for maximizing the benefit of lucerne production, we created a scenario where film replacement stopped after Year 6, and the yields remained unchanged to reduce the investment cost of film mulch and increase its legacy effect in terms of cumulative net income in Years 7–9 (**Figure 9C**). In this scenario, the cumulative net income difference between the M1 and M0 treatments (M1–M0) peaked three years later in Year 8 (5,023 RMB Yuan ha^–1^), with an increase of 1,113 RMB Yuan ha^–1^ (**Figure 9D**).

## 4 Discussion

### 4.1 The yield-increasing effect of film mulch and P application on lucerne

In semiarid areas, film mulch is widely used in crop production to enhance soil water supply capacity and increase yield (Zhu et al., 2018; Sun et al., 2020; Luo et al., 2022; Zhang et al., 2022). Consistent with previous studies (Jia et al., 2006b; Li et al., 2007; Wang et al., 2015), film mulch significantly increased the 0–100 cm soil water content, promoting seedling emergence rate, branch number per plant, plant height, shoot number, and biomass in lucerne in Years 1–5, and advanced the peak annual average forage yield by three years, relative to the M0 treatment. Film mulch can also promote lucerne growth and productivity by increasing surface soil temperature and soil nutrient availability (Jia et al., 2006a; Jia et al., 2006b). Lucerne is a perennial forage legume that can be used for long periods once established (Ali et al., 2021). While previous studies have demonstrated the short-term (2–3 years) benefits of film mulch on lucerne production (Jia et al., 2006b; Li et al., 2007; Wang et al., 2015), this study reveals a novel finding: the yield-increasing effect of film mulch gradually diminishes with lucerne stand years, disappearing completely in Years 6–8 and reducing production in Year 9. This insight has not been reported previously and is valuable for optimizing the long-term benefits of film mulch in sustainable lucerne production.

In semiarid environments with alkaline calcareous soils, soil P bioavailability is often limited, and continuous lucerne cultivation can deplete soil available P (Jiang et al., 2006; Wang et al., 2021). Increasing soil available P can accelerate lucerne growth, increasing plant height and biomass (Berg et al., 2005; Fan et al., 2011). However, in our study, we found that P application did not significantly increase lucerne forage yield in Years 1 and 2 due to the slow action of P fertilizer and its application in ridges. From Year 3, P fertilization alone significantly increased soil available P and lucerne forage yield, as reported elsewhere (Berg et al., 2007; Fan et al., 2011). Interestingly, the availability of soil P did not affect the growth of film-mulched lucerne after Year 6, despite the increase in soil available P and shoot P contents with increasing lucerne stand age and P application rates. Thus, no yield-increasing effect of P application was observed in film-mulched treatment after Year 6, suggesting that other nutrients might influence the yield response to P application in conjunction with film mulch. Fan et al. (2011) reported that four years of continuous N + P + organic fertilizer significantly increased lucerne forage yield in the years that P application alone did not. Therefore, it is important to consider incorporating additional elements alongside continuous P fertilization and film mulch to ensure sustained high lucerne yield and nutrient balance.

### 4.2 Soil N availability

Lucerne is a perennial legume that can fix atmospheric N in the atmosphere. Its strong N fixation ability and weak dependence on soil N can increase soil N content due to its large biomass (Yuan et al., 2016). Thus, film mulch significantly increased soil TN due to its pronounced yield-increasing effect in Years 1–5. However, the effectiveness of N fixation in lucerne could be constrained on the semiarid Loess Plateau, with limited rhizobia inoculation and scarce precipitation (Fan et al., 2016). In addition, lucerne’s ability to fix atmospheric N decreases with stand age (Duan et al., 2016). Therefore, while continuous planting of lucerne will increase the soil TN content, it results in low soil N availability and a gradual decline in soil inorganic N content over time (Jia et al., 2006a; Yuan et al., 2016). Fan et al. (2016) reported that N application significantly increased lucerne forage yield, with older stands (6–9 years) exhibiting greater yield increases than younger stands (2–5 years). Therefore, we speculated that soil N availability affects the sustainability of the yield-increasing effect of film mulch. Surprisingly, in the current study, despite the low soil N availability (average 3.6 mg kg^–1^) in all treatments, film mulch did not decrease soil inorganic N. Instead, it increased soil inorganic N in Years 4–6 and 9 due to the effective inhibition of ammonia volatilization (Guo et al., 2019). Furthermore, the correlation between soil IN and forage yield changed from positive to negative as the yield-increasing effect of film mulch diminished over time. This shifting relationship suggests that factors other than soil N availability might contribute to the observed decline in the yield-increasing effect of film mulch in the later years of lucerne cultivation.

### 4.3 Soil K availability

Soil K availability positively correlated with lucerne production in previous research (Kou et al., 2011; Feng et al., 2022). In northern China, soil available K and slowly available K concentrations are generally >100 and >600 mg kg^–1^, respectively, indicating that K is not a limiting factor for plant growth (Sun et al., 2014). Once plants have depleted the available K in soils, the slowly available K can be released to fulfill plant nutrient requirements (Flores et al., 2021). Film mulch can enhance soil K availability by increasing soil water content and microbial activity (Zhu et al., 2018). In the present study, film mulch and P application increased lucerne yield in the first four years without significant changes in soil available K due to enhanced K uptake (**Figure S2**). Lucerne has a high K demand and, unlike N, the K needed for growth must be sourced from the soil. Thus, the higher the lucerne forage yield, the more soil K is absorbed. In our study, lucerne in the M1 treatment removed 306 kg ha^–1^ more K from the soil than the M0 treatment in Years 1–5 (**Figure S2**). On the semiarid Loess Plateau, where low soil water availability and high soil cation exchange capacity significantly affect soil K^+^ mobility and absorption by lucerne (Lu, 2003; He et al., 2014), the slowly available K in soil may become inadequate to meet K absorption, decreasing soil available K over time (Lu, 2003; He et al., 2014). Moreover, soil available K was negatively correlated with soil water content and AP after Year 6, indicating that the improvements in water and P availability did not translate into increased soil K availability. Therefore, film mulch and P application significantly reduced soil K availability from the 6th year onwards.

Stand density is a critical factor affecting lucerne production and persistence. A strong positive correlation was observed between forage yield and stand density (**Figure 8**), with the decline in lucerne density coinciding with the disappearance of the yield-increasing effect of film mulch. Compared with N and P, soil K availability was more closely related to lucerne density (Berg et al., 2007). Soil K availability is crucial to lucerne’s overwintering ability and tolerance to frequent harvesting, and K fertilization can increase carbohydrate levels in the root and crown of lucerne, improve disease resistance, and prolong lucerne growth (Jungers et al., 2019). As lucerne stand ages, the yield-increasing effect of K fertilizer improves (Berg et al., 2007). Therefore, the decrease in lucerne density observed in the mulching treatment was more closely related to soil K availability rather than N and P availability (**Figure S5**). Studies have shown that young lucerne exhibits high forage yields, while older lucerne experiences poor plant persistence and low yields due to K deficiency (Lissbrant et al., 2010; Berg et al., 2018). Moreover, K deficiency may lead to more severe root rot in the mulching treatment (Jungers et al., 2019), resulting in decreased density and the disappearance of yield-increasing effects. While we noted this issue at harvest, we did not collect accurate data on root rot as it would have required digging up the plants, causing irreparable damage to the field plots. Thus, we speculate that the decrease in soil available K content under film mulch treatment leads to a decrease in lucerne density, ultimately resulting in the disappearance of the yield-increasing effect of film mulch.

Plants grown in nutrient-deficient soils often exhibit low shoot nutrient concentrations (Güsewell et al., 2003; Jungers et al., 2019). Since post-cut fertilization is not common in the semiarid Loess Plateau, shoot nutrient concentrations in the last lucerne cut (the second cut in the present study) better reflect its nutritional status and are closely associated with lucerne productivity. In our study, film mulch produced significantly higher shoot K concentrations in young lucerne during the second cut than in the no-mulch treatment. However, when the yield-increasing effect of film mulch disappeared, mulching produced significantly lower shoot K concentrations than no mulching, indicating that lucerne becomes more susceptible to K limitations in the mulching treatment. Moreover, lucerne growth under mulching was not restricted by N or P, as film mulch did not affect shoot N and P concentrations during the second cut in Years 6–9. We found that the yield-increasing effect of mulching positively correlated with differences in shoot K concentrations between mulched and non-mulched treatments during the second cut, whereas differences in shoot N and P concentrations did not show the same correlation (**Figure S6**). Thus, we speculate that the decline in the yield-increasing effect of film-mulched lucerne was associated with the decline in soil K availability rather than N and P availability. Nevertheless, further field research is needed to conclusively establish soil K availability as the primary factor affecting the yield-increasing effect of film mulch. Our findings also highlight the significance of maintaining soil nutrient balance in high-yielding lucerne pastures.

### 4.4 Soil organic C mineralization

The mineralization of SOC is an important indicator for measuring matrix availability and soil microbial activity and is directly related to soil nutrient release and plant growth (Yang et al., 2022). Soil microorganisms play a vital role in SOC mineralization, and their abundance and activity directly affect the SOC mineralization rate. In Years 2–5, film mulch significantly increased soil LFOC and SOC due to increased organic inputs through plant residues and rhizodeposits, stimulating the mass reproduction of soil microorganisms and increasing soil MBC (Jia et al., 2006a). Moreover, the improved soil hydrothermal conditions under film mulching created favorable conditions for soil microorganism growth and reproduction (Zhu et al., 2018), significantly increasing soil MBC. This, in turn, increased SOC mineralization and soil nutrient availability, improving lucerne growth. In Years 7–9, the yield-increasing effect of film mulch completely disappeared, despite the high SOC and appropriate soil hydrothermal conditions remaining under the film mulch. While film mulch did not significantly affect soil LFOC content during this period, we speculate that it increased soil MBC content despite significantly reducing soil MBC and SOC mineralization. Moreover, a lower soil MBC was associated with reduced lucerne shoot number growth, indicating that film mulch inhibited soil microorganism activity and lucerne growth after Year 6.

Several studies have demonstrated that deficiencies in mineral elements such as N, P, and K in the soil substrate inhibit soil microorganism growth and activity (Chandra et al., 2002; Fisk et al., 2015). Interestingly, we found that soil MBC content was positively correlated with the soil available K but not soil inorganic N or available P content when the yield-increasing effect of film mulch disappeared, suggesting that soil K may be the main factor affecting soil microorganism activity in the M1 treatment. The increase in soil K availability do not significantly affect soil microbial activity and microbial community composition in K-rich soils (Mori et al., 2019; Chen et al., 2020); however, when soil K is deficient, K addition significantly changed soil bacterial and fungal diversity and community composition, enhanced SOC mineralization, and promoted plant growth (Chandra et al., 2002; Li et al., 2021). In our study, adding K enhanced SOC mineralization in the M1 treatment but not in the M0 treatment (except for P3). Hence, we speculate that low soil K availability under film mulching suppresses soil microorganism activity, inhibits plant growth, reduces lucerne density, and ultimately decreases the yield-increasing effect.

### 4.5 Soil water storage

Lucerne has a high water-consuming rate and large water demand due to its high biomass and deep root system (Huang et al., 2018; Wang et al., 2021). As reported elsewhere (Jia et al., 2006b; Fan et al., 2016), film mulch and P application significantly enhanced deep-water use in Years 3–6 due to increased forage yield. Studies have attributed the decline in forage yield to a reduction in SWS (Huang et al., 2018; Wang et al., 2021). In this study, the yield-increasing effect of film mulch completely disappeared in Years 7–9, but film mulch did not reduce SWS in the 0–500 cm layer and significantly increased the top 300 cm soil water content due to its efficient ability to collect precipitation and reduce soil evaporation (Li et al., 2007). Notably, the M1 treatment had a similar soil water status in the 0–300 cm and 0–500 cm layers at the beginning of the growing season in Year 9 to that in Year 5, despite Year 9 receiving 40% more precipitation than Year 5 (416 mm c.f. 297 mm, respectively). However, film mulch significantly increased lucerne yield in Year 5 but showed a decreased yield in Year 9. Moreover, the correlation between lucerne yield and the top 100 cm soil water content changed from positive to negative after Year 6, indicating that soil desiccation was not the leading cause of the disappearing yield-increasing effect of film-mulched lucerne.

Lucerne–crop rotations are prevalent on the Loess Plateau due to lucerne’s significant contribution to soil quality enhancement (Ali et al., 2021). However, the soil water deficit and resultant deep soil drying in perennial lucerne grasslands are not conducive to good soil water balance and subsequent crop growth (Wang et al., 2008). In our study, as the lucerne plants grew, their roots probably extended deeper into the soil to access water from deeper layers, gradually depleting soil water in the 100–500 cm layer in all treatments from Years 1–7, as reported elsewhere (Fan et al., 2016; Yuan et al., 2016). However, in Years 8–9, with declining forage yield and increased precipitation, SWS in the 0–300 cm soil layer increased in all treatments. Notably, the M1 treatment had a greater amplitude and depth of soil water recovery in the 0–300 cm soil profile than the M0 treatment. Given that crops typically use soil water from 0–300 cm in this region (Cheng et al., 2005), the accelerated and deeper water recovery under film mulch could benefit subsequent crop growth. This study demonstrates that film mulch can alleviate soil water deficits, improve deep-water recovery, and enhance water use efficiency after Year 6. However, P application did not contribute to the acceleration of soil water recovery in later years, consistent with previous findings (Fan et al., 2011; Fan et al., 2016).

### 4.6 Optimum lucerne planting duration for increased forage production

In the pursuit of maintaining high land productivity, it is essential to determine the optimal utilization period for lucerne cultivation. We used the annual cumulative average of net income of a lucerne pasture as an index to identify reasonable production utilization years. Considering the changes in annual cumulative average of net income, our findings showed that the reasonable production years for maximizing income were five years under film mulch and eight years without mulch, with P application levels of P1 and P3, respectively (**Figure 9B**). However, based on our observations, we suggest extending the lucerne production years under film mulch for the following reasons: (1) soil water use by lucerne is an important factor affecting the number of productive years (Cheng et al., 2005; Wang et al., 2008), and the soil water at 0–300 cm depth was quickly restored under film mulch from Year 6; (2) the average yield in mulching in Years 6–8 reached 8,886 kg ha^–1^, surpassing the National Lucerne Industry Development Plan (2016–2020) development target of 7,500 kg ha^–1^ (http://www.moa.gov.cn/nybgb/2017/dyiq/201712/t20171227_6129812.htm), and the production value remained high; (3) farmers are more inclined to extend the production years of lucerne in semiarid areas due to establishment difficulties and the low forage yield during the first two years; and (4) The plastic film can be used for 2–3 years to reduce its usage and residue pollution, and studies have shown that re-used mulch produced equal yields for temperature-sensitive crops and higher yields for temperature-insensitive crops compared to no mulch (Liu et al., 2009; Zhang et al., 2022). Because re-used mulch had a lower capacity to retain soil water content and increase soil temperature than new film but still higher than no mulch (Liu et al., 2009; Zhang et al., 2022). This suggests that plastic film could be reused for another three years after Year 5, thus reducing costs. Based on these considerations, we propose an optimal lucerne production scenario: cultivating lucerne with film mulching and P application (9.7 kg ha^–1^) for five years and reusing the plastic film for an additional three years after Year 5. This strategic approach will expand the income-increasing effect of film mulch in the first five years, maximize economic benefits, and facilitate soil water recovery in semiarid areas.

## 5 Conclusions

This study presents novel insights into the dynamics of film mulch’s impact on lucerne pasture, shedding light on important trends and factors that influence forage yield and sustainability. This study is the first to report that the yield-increasing effect of film mulch on lucerne pasture gradually decreases with stand age and disappears after five years. Continuous lucerne production with film mulching for five years did not reduce soil N and P availability but significantly reduced soil MBC, SOC mineralization, K availability, lucerne density, and shoot K concentration. Phosphorus application under film mulching significantly increased lucerne forage yield before Year 6 but had no such effect subsequently. Moreover, film mulch significantly promoted soil water recovery after the yield-increasing effect disappeared. Thus, the disappearance of the yield-increasing effect of film-mulched lucerne was unrelated to soil moisture, N availability, or P availability, but closely related to K availability. Future research should focus on implementing K fertilizer strategies to ensure the sustained yield-increasing effect of film mulching and verifying the scenario of retaining used film mulch after year 6 could be a valuable approach to further increase forage production and associated income while avoiding soil water depletion.

## Data availability statement

The original contributions presented in the study are included in the article/**Supplementary Material**. Further inquiries can be directed to the corresponding authors.

## Author contributions

MK: Conceptualization, Data curation, Formal Analysis, Writing – review & editing, Investigation, Writing – original draft. YG: Data curation, Formal Analysis, Investigation, Writing – review & editing. CH: Data curation, Formal Analysis, Investigation, Writing – review & editing. XS: Data curation, Formal Analysis, Investigation, Writing – review & editing. JK: Data curation, Formal Analysis, Investigation, Writing – review & editing. KS: Formal Analysis, Writing – review & editing. FL: Formal Analysis, Writing – review & editing, Conceptualization, Data curation, Funding acquisition, Project administration. ZY: Conceptualization, Formal Analysis, Writing – review & editing.
