# Supplementary material for: The prolonged effect of film mulch and P application on lucerne forage yield in a semiarid environment
Source: Front Plant Sci. 2023 Dec 11;14:1331704. doi: 10.3389/fpls.2023.1331704 (PMC10749361; doi:10.3389/fpls.2023.1331704)
Supplement: Supplementary file 1 [file DataSheet_1.docx]

Supplementary Material

# Supplementary Figures and Tables

**Table S1**Design P fertilizer application rate and actual application amount per year during the experiment (2011–2019).

|  | Design P levels (kg ha^–1^) | Annual actual phosphorus application (kg ha^–1^) | | | | | | | | | |
| --- | --- | --- | --- | --- | --- | --- | --- | --- | --- | --- | --- |
|  |  | 2011 | 2012 | 2013 | 2014 | 2015 | 2016 | 2017 | 2018 | 2019 | Mean |
| P0 | 0 | 0 | 0 | 0 | 0 | 0 | 0 | 0 | 0 | 0 | 0 |
| P1 | 17 | 5.31 | 15.8 | 11.4 | 9.73 | 7.82 | 8.26 | 9.74 | 9.36 | 9.48 | 9.7 |
| P2 | 34 | 10.3 | 30.6 | 22.9 | 19.5 | 15.6 | 16.5 | 19.5 | 18.7 | 19 | 19.2 |
| P3 | 51 | 15.7 | 45.9 | 34.3 | 29.2 | 23.5 | 24.8 | 29.2 | 28.1 | 28.4 | 28.8 |

**Table S2** Net income (RMB Yuan ha^–1^) for no film mulch (M0) and film mulch (M1) at four P levels [0 (P0), 9.7 (P1), 19.2 (P2), and 28.8 (P3) kg P ha^−1^ year^–1^] in Gansu Province, China.

| Treatment | | Annual input **^a^**  (RMB Yuan ha^–1^) | Net income (RMB Yuan ha^–1^) **^b^** | | | | | | | | | |
| --- | --- | --- | --- | --- | --- | --- | --- | --- | --- | --- | --- | --- |
|  |  |  | 2011**^c^** | 2012 | 2013 | 2014 | 2015 | 2016 | 2017 | 2018 | 2019 | 2011–2019 |
| M0 | P0 | 0 | –565 | 1,759 | 2,476 | 2,982 | 2,937 | 2,707 | 3,102 | 2,827 | 2,033 | 20,258 |
|  | P1 | 15.3 | –579 | 1,687 | 3,615 | 5,012 | 3,634 | 3,496 | 3,826 | 3,922 | 2,853 | 27,466 |
|  | P2 | 30.6 | –597 | 1,870 | 4,044 | 6,414 | 4,366 | 4,390 | 4,493 | 4,509 | 3,164 | 32,652 |
|  | P3 | 45.9 | –613 | 1,779 | 4,927 | 5,496 | 4,999 | 5,108 | 5,334 | 4,806 | 3,511 | 35,347 |
| M1 | P0 | 1,389 | –1,751 | 4,110 | 3,490 | 4,787 | 3,344 | 2,990 | 2,667 | 2,490 | 918 | 23,045 |
|  | P1 | 1,404 | –1,776 | 5,004 | 5,727 | 5,856 | 5,792 | 3,562 | 2,981 | 2,746 | 895 | 30,787 |
|  | P2 | 1,420 | –1,770 | 5,293 | 4,803 | 5,983 | 5,335 | 3,154 | 2,875 | 3,100 | 813 | 29,585 |
|  | P3 | 1,435 | –1,789 | 4,807 | 4,990 | 5,406 | 3,645 | 3,367 | 3,081 | 3,290 | 1,439 | 28,235 |
| *p*-values of ANOVA in randomized blocks of split-plot design | | | | | | | | | | | | |
| M | |  | **<0.001** | **0.046** | **0.004** | 0.396 | 0.104 | 0.213 | **0.007** | **0.014** | **0.003** | 0.187 |
| P | |  | 0.156 | 0.539 | **0.004** | **0.013** | 0.173 | 0.107 | **0.005** | **<0.001** | 0.118 | **0.002** |
| M × P | |  | 0.894 | 0.616 | 0.199 | 0.269 | 0.213 | 0.195 | **0.037** | **0.08** | 0.424 | 0.108 |

^a^ Farmers in the semiarid area of the Loess Plateau do not consider labor costs in traditional lucerne production, so the annual inputs only included the money spent on purchasing phosphate fertilizer and plastic film, forming ridges, and covering film on the ridges. The M1 treatment had 1,389 RMB Yuan ha^–1^ higher annual investment than the M0 treatment due to purchasing plastic film and the labor cost of forming ridges and covering film on the ridges. The price of phosphate fertilizer was 1.125 Yuan kg^–1^.

^b^ Net income equals income minus inputs. Income is forage yield multiplied by lucerne price. The local price of lucerne forage was 0.5 RMB Yuan kg^−1^.

^c^ The costs of buying lucerne seeds (35 RMB Yuan kg^−1^) and urea (2.0 RMB Yuan kg^−1^) were only added to the inputs in the first year.


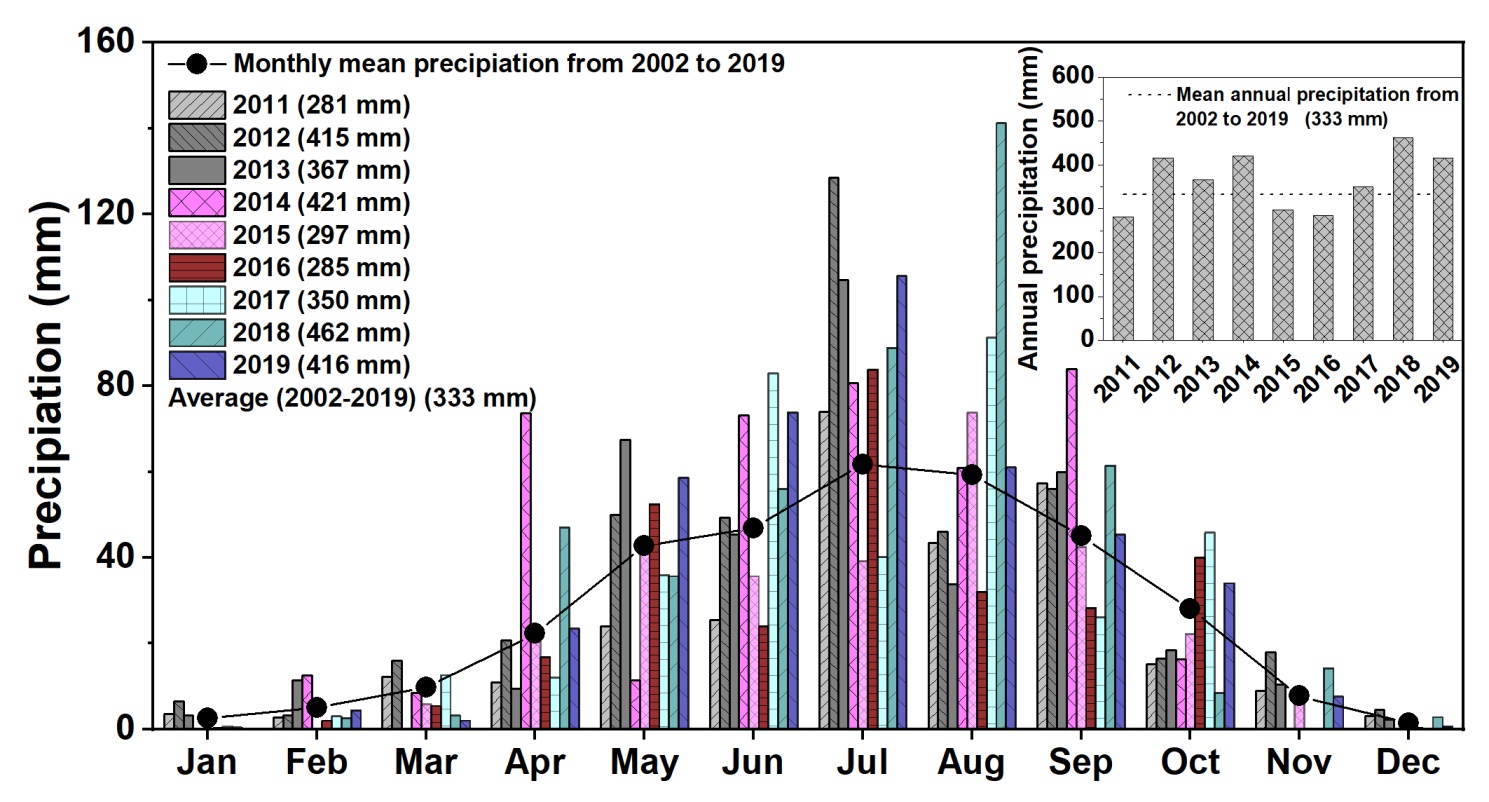


**Fig. S1** Monthly precipitation each year and long-term monthly mean precipitation (2002–2019) at the Semi-Arid Ecosystem Research Station of Lanzhou University, Zhonglianchuan, Gansu, China.


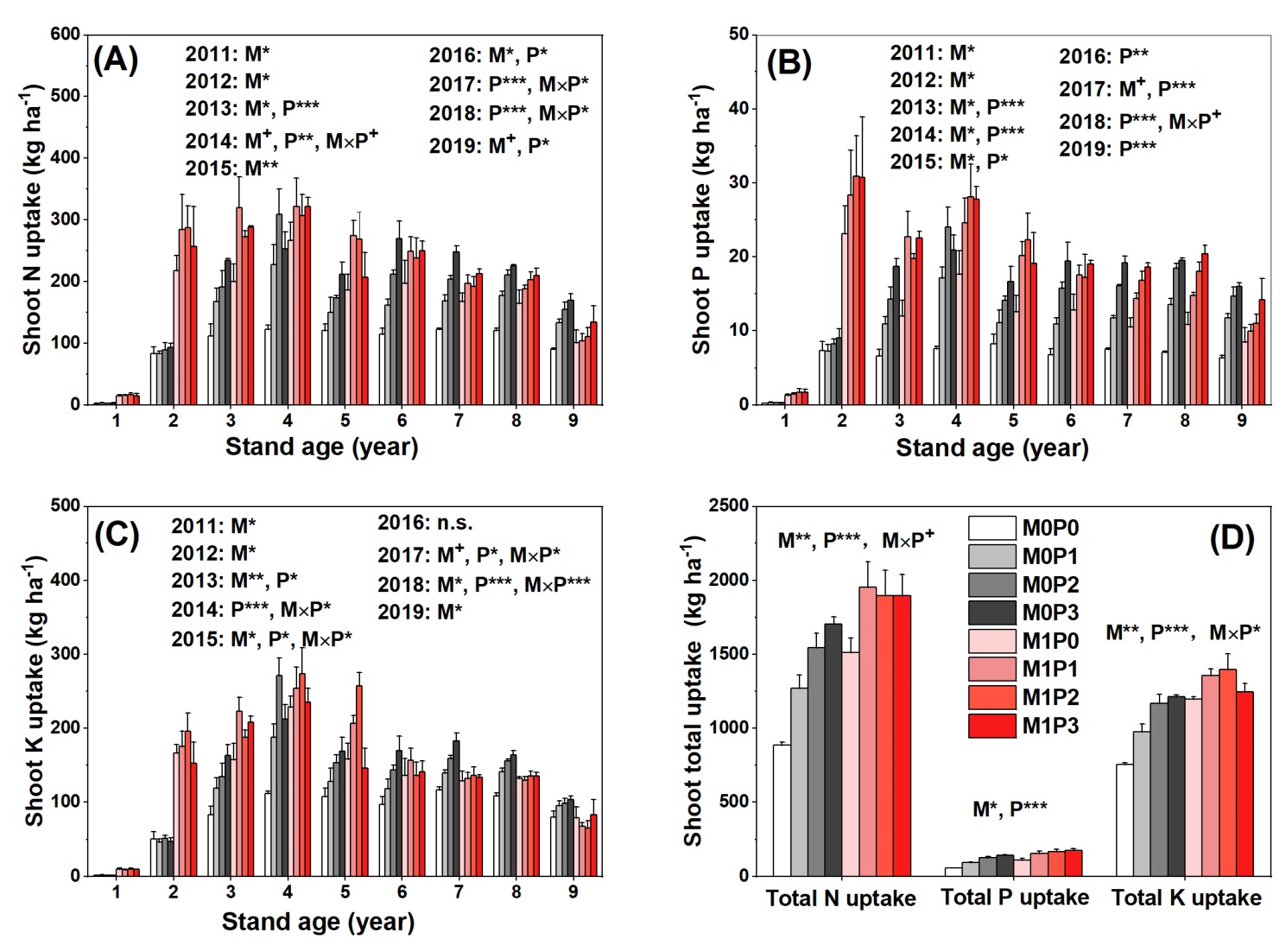


**Fig. S2** Dynamic changes in (A, B, C) annual and (D) total lucerne shoot N, P, and K uptake for no film mulch (M0) and film mulch (M1) at four P levels [0 (P0), 9.7 (P1), 19.2 (P2), and 28.8 (P3) kg P ha^−1^ year^–1^]. *** *p* ≤ 0.001, ** *p* ≤ 0.01, * *p* ≤ 0.05, and + *p* ≤ 0.1. Omitted contrasts and those shown with an abbreviation (n.s.) were not significant (*p* > 0.1). Data are mean ± standard error (n = 3).


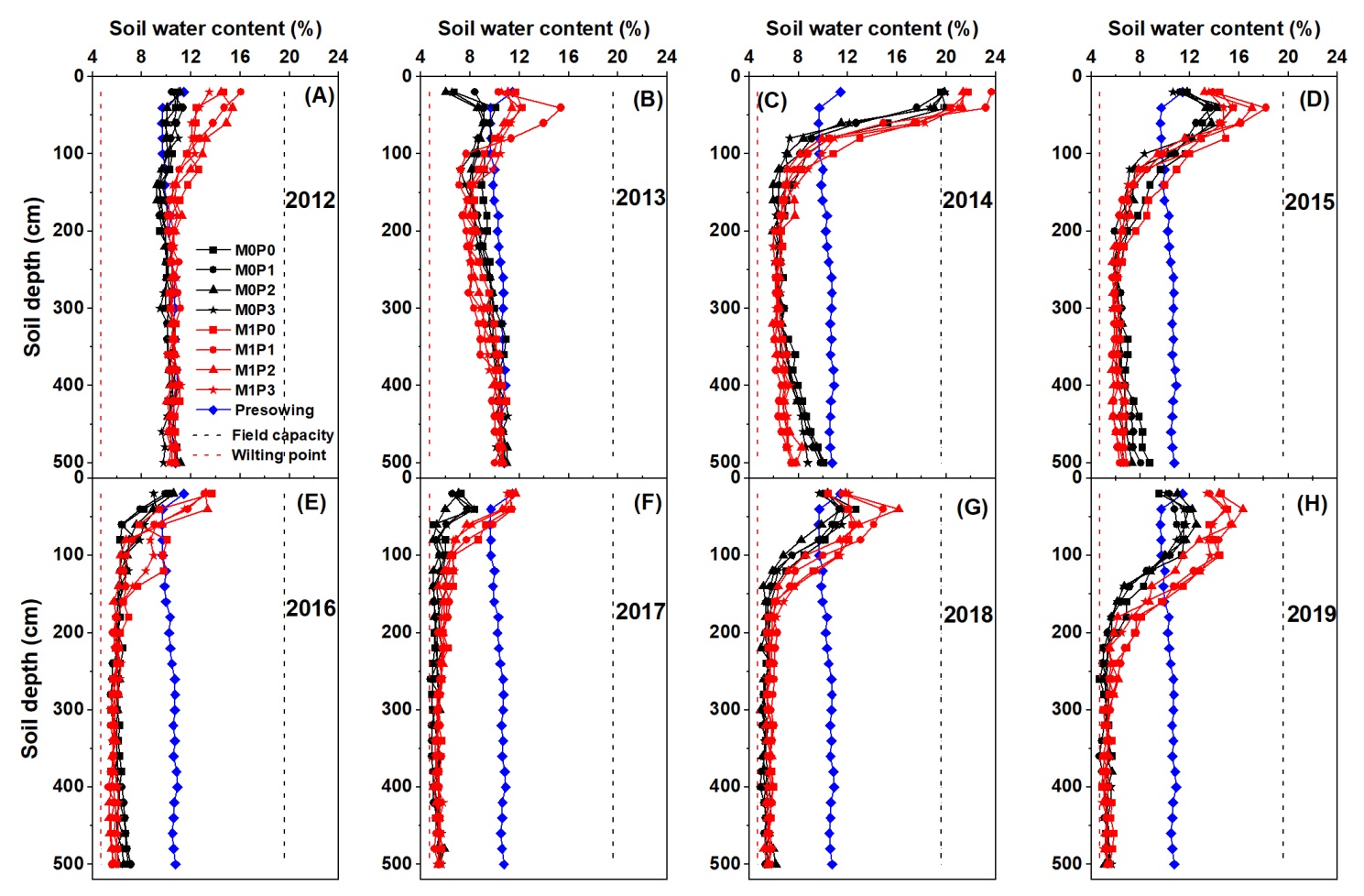


**Fig. S3** Profile of gravimetric soil water content at 20 cm increments in the upper 5 m of the soil profile in April at the beginning of the growing season from 2012 to 2019 in no film mulch (M0) and film mulch (M1) treatments at four P levels [0 (P0), 9.7 (P1), 19.2 (P2), and 28.8 (P3) kg P ha^−1^ year^–1^]. The blue line is the soil water content pre-sowing (June 2011). The black and red dotted lines indicate the soil water content at field capacity and permanent wilting point, respectively.


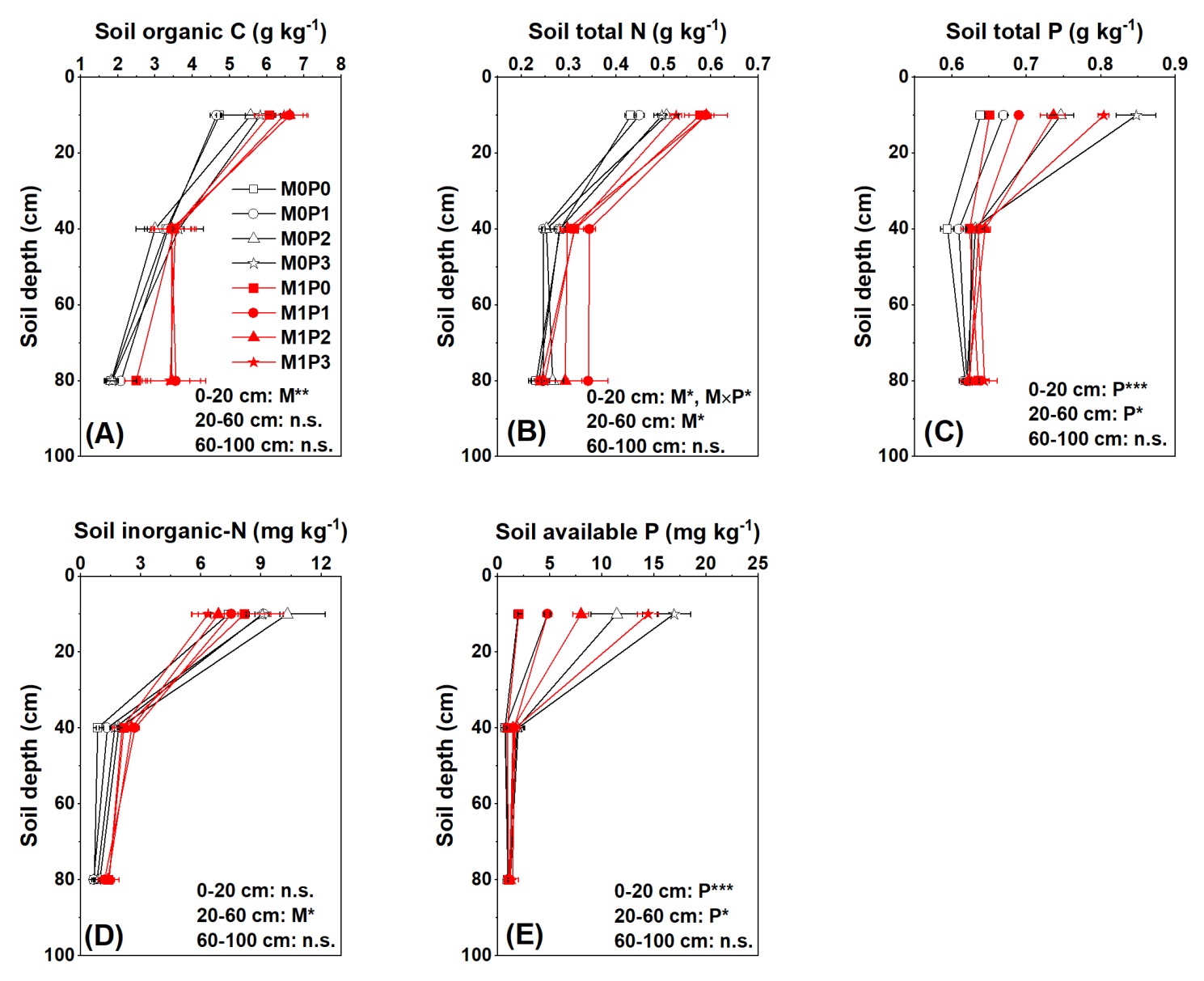


**Fig. S4** Soil (A) organic C, (B) total N, (C) total P, (D) inorganic N, (E) available P contents in the 0–20, 20–60, and 60–100 cm soil profiles for no film mulch (M0) and film mulch (M1) at four P levels [0 (P0), 9.7 (P1), 19.2 (P2), and 28.8 (P3) kg P ha^−1^ year^–1^] in April 2019. *** *p* ≤ 0.001, ** *p* ≤ 0.01, * *p* ≤ 0.05, and + *p* ≤ 0.1. Omitted contrasts and those shown with an abbreviation (n.s.) were not significant (*p* > 0.1). Data are mean ± standard error (n = 3).


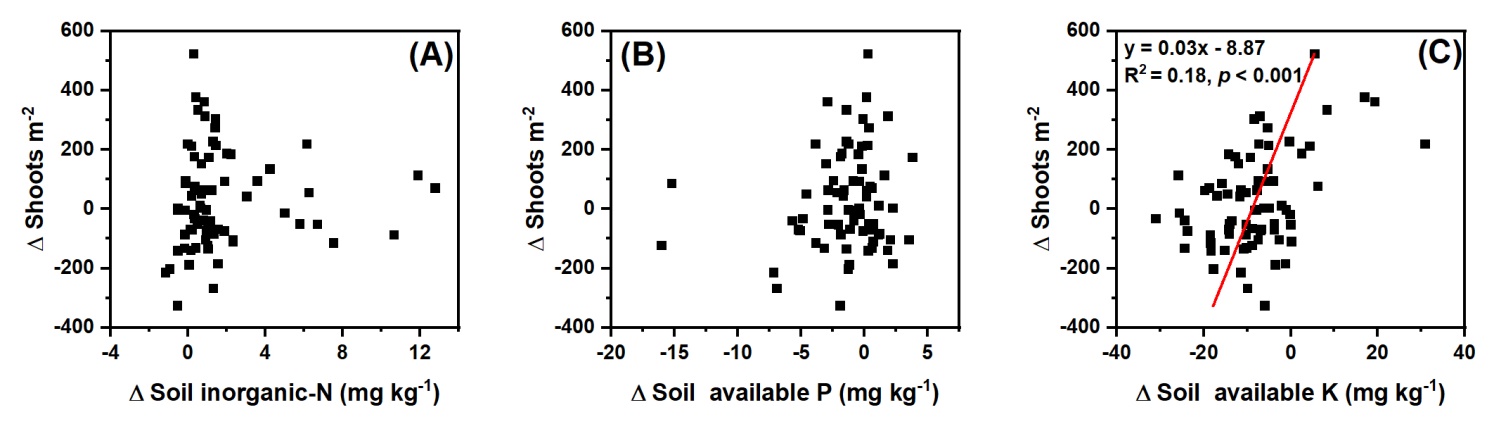


**Fig. S5** Regressions for the difference in the number of lucerne shoots per unit area (∆ shoot) between M1 and M0 and the difference in (A) soil inorganic N, (B) available P, and (C) available K between M1 and M0.


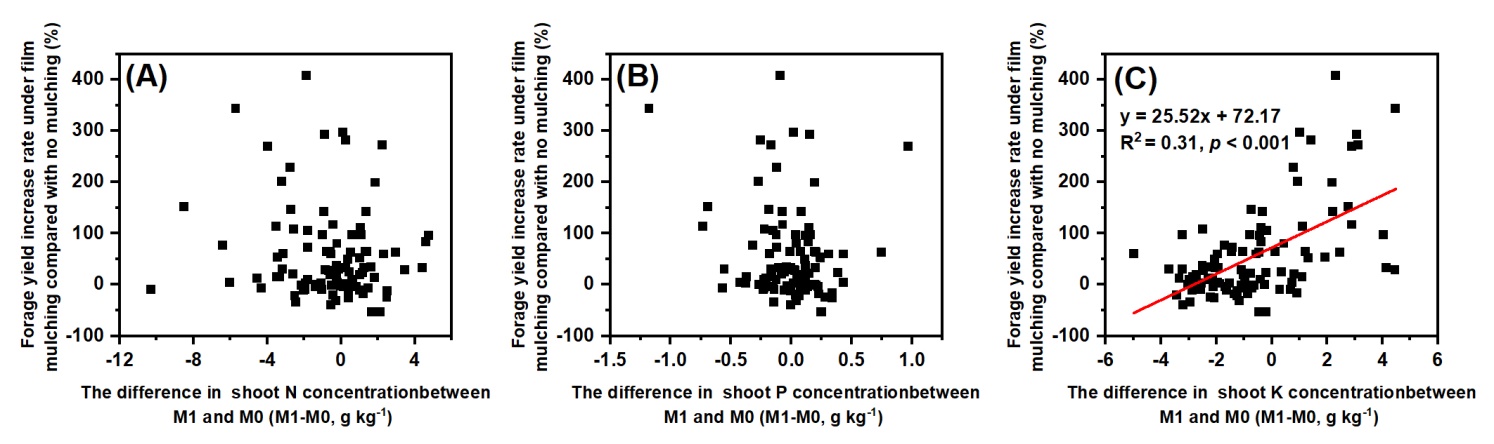


**Fig. S6** Regressions for the yield-increasing effect of mulching and difference in lucerne (A) shoot N, (B) shoot P, and (C) shoot K concentrations between M1 and M0 (M1–M0) in the second cut.
